# Prevalence of Mitral Valve Prolapse Among Individuals with Pectus Excavatum: A Systematic Review and Meta-Analysis

**DOI:** 10.3390/diagnostics14222488

**Published:** 2024-11-07

**Authors:** Andrea Sonaglioni, Antonino Bruno, Alessio Polymeropoulos, Gian Luigi Nicolosi, Michele Lombardo, Paola Muti

**Affiliations:** 1 Division of Cardiology, IRCCS MultiMedica, 20123 Milan, Italy; michele.lombardo@multimedica.it; 2 Laboratory of Innate Immunity, IRCCS MultiMedica, 20138 Milan, Italy; antonino.bruno@multimedica.it; 3 Laboratory of Immunology and General Pathology, Department of Biotechnology and Life Sciences, University of Insubria, 21100 Varese, Italy; 4 Fondazione IRCCS Istituto Nazionale dei Tumori, 20133 Milan, Italy; alessio.polymeropoulos@istitutotumori.mi.it; 5 Division of Cardiology, Policlinico San Giorgio, 33170 Pordenone, Italy; gianluigi.nicolosi@gmail.com; 6 Department of Biomedical, Surgical and Dental Sciences, University of Milan, 20122 Milan, Italy; pmuti26@gmail.com; 7 IRCCS MultiMedica, 20138 Milan, Italy

**Keywords:** pectus excavatum, Haller index, mitral valve prolapse, prevalence, meta-analysis

## Abstract

**Background**: During the last decades, a small number of studies reported a wide range of variability in the estimated prevalence of mitral valve prolapse (MVP) among individuals with pectus excavatum (PE). The present systematic review and meta-analysis has been primarily designed to summarize the main findings of these studies and to estimate the overall prevalence of MVP among PE individuals. **Methods**: All imaging studies assessing the prevalence of MVP in PE individuals vs. healthy controls, selected from PubMed and EMBASE databases, were included. The risk of bias was assessed by using the National Institutes of Health (NIH) Quality Assessment of Case–Control Studies. Events (presence of MVP) and nonevents (absence of MVP) in PE individuals and control groups were recorded. The main outcome was the measure of odds ratio (OR) for MVP presence pooled with 95% confidence intervals, using a fixed-effects model. **Results**: The full texts of eight studies with 303 PE patients (mean age 25.7 yrs) and 498 healthy controls (mean age 31 yrs) were analyzed. Three studies assessed MVP prevalence in children and early adolescents, whereas the remaining five studies examined PE adults. The prevalence of MVP in PE individuals and healthy controls was 40.6% and 12.8%, respectively. In the pooled sample, the OR for MVP presence was significantly higher in PE individuals compared to controls (OR = 5.80, 95%CI = 3.83–8.78, Z = 8.30, *p* < 0.001). Subgroup analysis revealed that MVP prevalence was approximately three-fold higher among PE children and early adolescents compared with PE adults. Overall, high consistency was observed in the pooled effect sizes, due to the low statistical heterogeneity among the included studies (I^2^ = 22.7%, *p* = 0.25). Egger’s test for a regression intercept gave a *p*-value of 0.07, indicating no publication bias. The sensitivity analysis supported the robustness of the results. **Conclusions**: PE individuals are nearly six times more likely to have MVP than controls. MVP prevalence is three-fold higher in PE individuals during childhood and early adolescence, compared to PE adults. Given the strong association between MVP and PE, MVP should be suspected in all individuals with anterior chest wall deformity.

## 1. Introduction

Pectus excavatum (PE) is the most common anterior chest wall deformity and is characterized by an inward displacement of the sternum and adjacent costal cartilage [1]. It occurs in an estimated 1 in 300–400 births, with male predominance (male-to-female ratio of 3:1) [2]. The anatomic compression from the chest wall may cause cardiopulmonary impairment, due to limitation of diastolic filling through mechanical compression of right-side heart chambers, and consequently limited exercise tolerance [3]. When severe, PE can be corrected surgically, with either the open or Ravitch procedure or the minimally invasive Nuss procedure [4,5]. PE is commonly quantified by the radiological Haller index (HI), calculated on an axial computed tomography (CT) scan by dividing the transverse diameter of the chest by the antero-posterior (A-P) distance between the posterior surface of the sternum and the anterior surface of the vertebral body, at the level of the deepest point of the sternal deformity [6]. An HI ≥3.25 is defined as the threshold for considering surgical repair of PE [7]. The mechanical compression caused by PE primarily involves the right ventricle (RV), due to its anterior location, thin wall and low pressure [8]. The RV develops a tubular configuration, with mid-ventricular retraction and rounded RV apex, and its systolic function is impaired [9]. The continuous mechanical stress exerted by the thoracic deformity has also been associated with the mechanical distortion of the mitral valve and the occurrence of mitral valve prolapse (MVP) in PE individuals [10]. On the other hand, MVP individuals are commonly found with various degrees of anterior chest wall deformity and/or thoracic skeletal abnormalities, such as PE, pectus carinatum, scoliosis, straight back syndrome and Marfan syndrome (MFS) [10].

During the last three decades, a small number of imaging studies have evaluated the relationship between MVP and PE, reporting a wide range of variability in the estimated MVP prevalence among PE individuals. The high variability of these results was likely related to sampling biases, small study samples and the not-univocal diagnostic criteria used for MVP diagnosis before and after the year 2000, respectively. Notably, the studies performed before the year 2000 included highly selective cohorts of individuals who were concerned about their cardiac status because of affected family members, who had a history of heart murmur or subtle symptoms and who were well known to the examining physician; moreover, the older studies used nonspecific echocardiographic criteria, including displacement of the anterior leaflet in the apical four-chamber view, to diagnose MVP [11,12]. Conversely, the studies performed after the year 2000 used more specific echocardiographic criteria, eliminating the use of the apical four-chamber view and thus reducing the false positive diagnoses [13]; these studies defined MVP as the systolic displacement of one or both mitral leaflets >2 mm above the mitral annulus plane, visualized from the parasternal long-axis view [14].

The present systematic review and meta-analysis has been primarily designed to summarize the main findings of the studies assessing MVP prevalence among PE individuals and to estimate the overall prevalence of MVP among PE individuals. The relevant pathophysiological mechanisms underpinning the association between PE and MVP will be discussed as well.

## 2. Materials and Methods

This systematic review and meta-analysis was performed according to the Preferred Reporting Items for Systematic Reviews and Meta-Analyses (PRISMA) guidelines [15] and was registered in the PROSPERO database (CRD42024566324).

### 2.1. Search Strategy

A comprehensive search of all imaging studies assessing the prevalence of MVP among individuals affected by PE vs. healthy controls with normal chest wall conformation was carried out by two independent reviewers (A.S. and M.L.) through July 2024, by using the PubMed, Scopus and EMBASE databases. The search strategy included the following terms: “pectus excavatum” OR “funnel chest” AND “mitral valve prolapse” AND “prevalence” AND “cardiac function” AND “echocardiography” OR “M-mode echocardiography” OR “two-dimensional (2D) echocardiography” AND “cardiovascular magnetic resonance (CMR)”. The search was limited to full-text articles published in English. There was no limitation on the time period.

### 2.2. Eligibility Criteria

All echocardiographic or CMR studies evaluating MVP prevalence in PE individuals vs. healthy controls were included. Conversely, imaging studies conducted on PE patients without data on MVP prevalence or without healthy controls, non-clinical articles, animal studies, duplicate articles, case reports, conference presentations, reviews, editorials, letters without data, and abstracts were excluded.

### 2.3. Study Selection and Data Extraction

Two reviewers (A.S. and M.L.) screened the databases according to the inclusion criteria and performed data extraction independently. Information concerning the following were independently collected by the two reviewers: (1) demographics (age and sex); (2) anthropometrics [body surface area (BSA)]; (3) degree of anterior chest deformity; (4) the prevalence of Marfan syndrome (MFS) among PE individuals; (5) electrocardiogram (ECG) findings; (6) conventional and innovative indices of cardiac morphology and function assessed by echocardiography or CMR; (7) prevalence of MVP and degree of mitral valve regurgitation (MVR); (8) concomitant valvulopathy, particularly tricuspid valve prolapse (TVP), or concomitant pericardial effusion; (9) the current medical treatment; (10) follow-up data, if any. A third author (G.L.N.) checked the extracted data for accuracy and resolved possible discrepancies between the reviewers.

### 2.4. Risk of Bias Assessment

**Figure 1:**
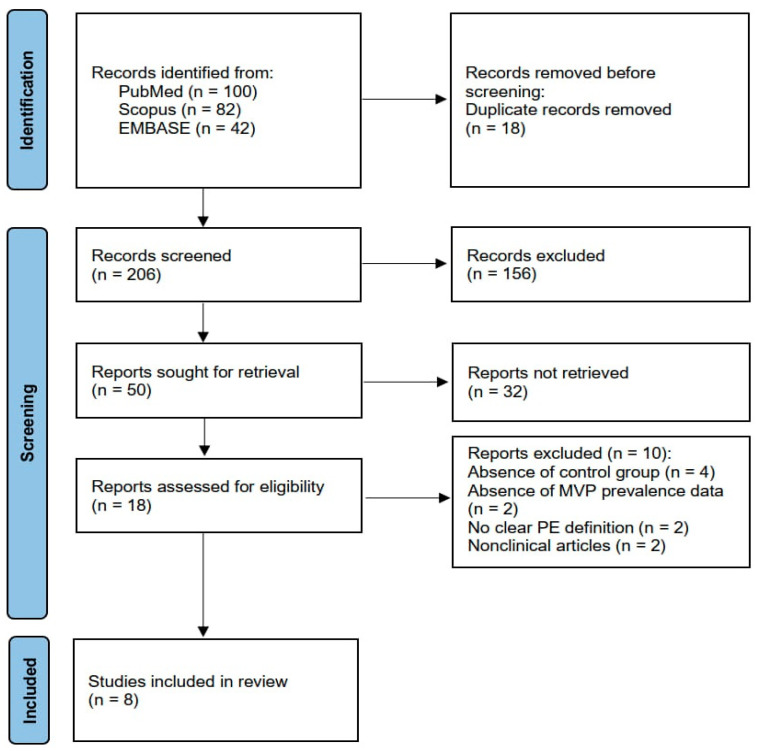
Flow diagram used for identifying included studies. MVP, mitral valve prolapse; PE, pectus excavatum.

The articles included in this systematic review and meta-analysis were assessed for risk of bias (RoB) using the National Institutes of Health (NIH) Quality Assessment of Case–Control Studies [16]. All studies were assigned a “yes”, “no” or “other” for each of the 12 criteria outlined in the appraisal tool. Then, by considering each criterion, the investigators evaluated the overall quality of the study and assigned an overall rating of “good” (met 9–12 criteria), “fair” (met 5–8 criteria) or “poor” (met 0–4 criteria) to each study. The quality rating was independently estimated by two authors (A.S. and G.L.N.). Disagreement was resolved by consensus. The PRISMA flow diagram used for identifying the included studies is depicted in Figure 1.

### 2.5. Statistical Analysis

The primary endpoint was to investigate the prevalence of MVP among individuals with PE. The events (MVP+) and nonevents (MVP−) in PE individuals and healthy controls extracted from the studies were entered into the dataset as raw numbers. The pooled data included number of events, number of nonevents, sample size of PE individuals and controls. The number of MVP cases (PE individuals) and controls and the odds ratios (ORs) for each study were calculated. A subgroup analysis was performed for evaluating the MVP prevalence in PE children vs. PE adults. The significance of pooled ORs estimates was tested by z tests. The homogeneity of the effect sizes across the studies was tested with the Cochrane’s Q test [17], and the magnitude of heterogeneity was expressed by the I^2^ (inconsistency) test [18]. If the homogeneity assumption was not violated (I^2^ < 25%), the ORs and weighted percentages were calculated using the fixed-effect model [19] based on inverse-variance meta-analysis [20]. Begg’s funnel plots and Egger’s test were employed to assess potential publication bias [21]. Finally, a sensitivity analysis was performed by investigating the effect of individual studies on the overall meta-analysis: the meta-analysis was re-estimated by omitting each study, sequentially, to determine the robustness of results [22]. The 95% confidence intervals (CIs) were calculated and two-tailed *p*-values below 0.05 were considered to be statistically significant. Statistical analysis was performed by using Comprehensive Meta-Analysis version 3.0 (Biostat, Englewood, NJ, USA) and the R programming language, version 4.3.2 (www.r-project.org/, accessed on 22 July 2024).

## 3. Results

### 3.1. Clinical Findings

The initial search yielded a total of 224 studies. Of those, 18 (8%) were removed as duplicates. Then, 206 studies (91.9%) were removed on the basis of the exclusion criteria. The evaluation of the full text of the remaining 18 studies (8%) resulted in 10 further exclusions (4.5%). A total of eight studies (3.6%) [23,24,25,26,27,28,29,30] were thus included in this systematic review and meta-analysis, totaling 303 PE individuals and 498 healthy controls without PE.

Clinical characteristics and main findings of the included studies are summarized in Table 1.

The included studies were published between 1990 and 2023. Two studies were performed in Italy and Turkey, and one in USA, Japan, Germany and Canada. Four studies (50% of total) [24,26,28,29] had a prospective design, whereas the remaining four (50% of total) [23,25,27,30] had a retrospective design. The mean age of PE individuals among the included studies was 25.7 yrs (range 7.6–55.8 yrs), while that of controls was 31 yrs (range 5.4–58.8 yrs). Three studies (37.5% of total) assessed MVP prevalence in children and early adolescents, whereas the remaining five studies (62.5% of total) examined PE adults. The percentage of males among PE individuals was significantly higher than healthy controls [68.2% (range 52–82%) vs. 66% (range 50–85%), *p* = 0.02)]. The average BSA of PE patients was significantly lower in comparison to controls [1.77 m^2^ (range 1.63–1.98 m^2^) vs. 1.83 m^2^ (range 1.63–2.1 m^2^), *p* < 0.001)].

Half of the studies used the conventional HI to quantify the severity of PE deformity, two studies involving children [23,27] defined the PE as a chest wall deformity of at least 2 cm from the surface of the anterior chest wall to the deepest point of the pectus, and one study [24] used the radiological sagittal–coronal index, while the remaining study performed by our research group [26] used the modified Haller index (MHI) as a noninvasive index of chest wall deformity. Hohneck A et al. [29] measured the degree of PE deformity using not only the conventional HI, but also different metrics, such as the Correction-Index and the Depression-Index. The average HI of PE patients among the included studies was 5.9 (range 2.9–8.3). A family history positive for PE was described in 35% of cases (range 26–44%). The pooled prevalence of MFS among the included studies was 0.5% (range 0–3.6%).

The majority of included studies (62.5% of total) [24,25,26,27,28] used 2D tranthoracic echocardiography for examining PE individuals, one study [23] used M-mode echo, and the remaining two studies [29,30] evaluated PE patients by CMR. Concerning MVP criteria, the great majority of studies (75% of total) defined MVP as a systolic billowing of one or both mitral leaflets >2 mm above the mitral annulus in the long-axis parasternal view for echocardiographic studies and in the end-systolic left ventricular (LV) three-chamber cine SSFP image for CMR studies, according to the revised diagnostic criteria [14,31]; on the other hand, the two studies of Park JM et al. [23] and Mocchegiani R et al. [24], which were performed before the year 2000, considered MVP to be present when there was >2 mm displacement of mitral leaflets in the parastemal long-axis or apical four-chamber views.

All the imaging studies included in this systematic review and meta-analysis provided a detailed evaluation of cardiac chambers cavity sizes, biventricular systolic function and degree of mitral valvulopathy. Three studies [26,29,30] described the main ECG findings observed among PE individuals vs. controls, whereas two authors [26,29] analyzed not only the conventional echocardiographic parameters but also the innovative myocardial deformation indices obtained in the two study groups.

### 3.2. ECG Findings

Compared to healthy controls, PE individuals were commonly found with increased prevalence of supraventricular extrasystoles and/or ventricular extrasystoles and nonspecific ST-segment and T-wave (NS-STT) abnormalities on resting ECG [26,29]. Our study group [26] detected isolated supraventricular and ventricular premature beats in 10.1% and 48.7% of PE individuals, respectively. Moreover, more than half of PE individuals (53.3% of total) were found having NS-STT abnormalities on resting ECG. In the series of Hohneck A et al. [29], supraventricular and ventricular extrasystoles were observed in 15% and 21% of PE patients, respectively. Abdulmonem L Hashem D et al. [30] found isolated supraventricular and ventricular premature beats in 3% and 13% of PE individuals, respectively. In our series [26], no case of atrial fibrillation (AF) was diagnosed in PE individuals, whereas Hohneck A et al. [29] and Abdulmonem L Hashem D et al. [30] detected AF in 2% and 3% of PE patients, respectively. No cases of malignant ventricular arrhythmias (VAs) nor sudden cardiac death (SCD) were reported by the included studies.

### 3.3. Conventional Indices of Biventricular Size and Function

The most frequent morphological abnormalities detected among PE individuals were the following: (1) the RV dilatation with a tubular configuration, characterized by midventricular retraction due to sternal compression and expansion of the apical diameter; (2) reduction in right ventricular ejection fraction (RVEF), RV emptying fraction and tricuspid annular plane systolic excursion (TAPSE); (3) impairment in both stroke volume (SV) and cardiac output (CO), despite a preserved left ventricular ejection fraction (LVEF); (4) a concomitant pericardial effusion, which was reported in 24% of PE individuals (29). With regard to LV volumes, our study group demonstrated that PE individuals had smaller LV internal dimensions than controls; conversely, the CMR study performed by Hohneck A et al. [29] found greater LV volumes in PE individuals vs. controls, whereas Abdulmonem L Hashem D et al. [30] did not detect a significant difference in LV volumes between PE individuals and controls.

### 3.4. Mitral Valve Assessment

In total, 123 out of 303 PE individuals (40.6%) and 64 out of 498 controls (12.8%) met the diagnostic criteria for MVP. The MVP rate increased with the severity of pectus deformity [23,26,27]. Concerning mitral valve morphology, two authors [25,29] demonstrated that patients with PE had significantly shorter scallop P2, longer scallop A2, shorter coaptation depths, and longer papillary muscle tethering lengths when compared with normal controls. MVR due to MVP was observed in 19.7% of PE individuals and 9.7% of controls, respectively. The average degree of MVR among PE individuals was mild (37.5% of cases) or mild-to-moderate (62.5% of cases), whereas no case of severe MVR was reported.

### 3.5. Myocardial Strain Parameters

Our study group found that all biventricular deformation parameters were severely reduced in PE individuals, in comparison to controls and to accepted reference values [32,33]; the impairment of myocardial strain parameters detected in PE individuals was particularly evident at the level of basal myocardial segments, which are in anatomical contiguity with anterior chest wall depression [26]. Differently from our findings, Hohneck A et al. [29] observed that LV deformation indices were not significantly different between PE individuals and healthy controls, whereas RV myocardial strain indices were significantly reduced in the PE group vs. controls.

### 3.6. Concomitant Cardiovascular Abnormalities

Several concomitant cardiovascular abnormalities were detected in PE individuals by the included studies. Acipayam A et al. [28] demonstrated significantly higher rates of cardiac malposition and congenital heart disease in the PE group compared with the control group. Hohneck A et al. [29] described mild pericardial effusion in 41% of PE patients, likely related to irritant effects by contact with the osseous/cartilaginous structures (contact zone). Abdulmonem L Hashem D et al. [30] demonstrated that MVP was associated with mitral annular disjunction (MAD) in 58% of PE individuals. Concomitant TVP was observed in 11% of PE individuals by Mocchegiani R et al. [24] and 25% of PE individuals by Acipayam A et al. [28]. In addition, our study group [26] and Acipayam A et al. [28] demonstrated that aortic root and ascending aorta diameters were significantly larger in PE individuals than controls. Interestingly, our study group [26] also described a significantly lower prevalence of the most relevant cardiovascular risk factors (smoking, hypertension, type 2 diabetes and dyslipidemia) among PE individuals in comparison to healthy controls.

### 3.7. Medical Treatment and Follow-Up Data

Data concerning the medical treatment prescribed to PE individuals are scanty. Three studies [26,29,30] reported that PE individuals were more frequently treated with β-blocker therapy than controls. Among the included studies, only Hohneck A et al. [29] provided follow-up data in PE individuals. Their cohort of patients underwent ablation (13.6%), medical treatment (mainly based on β-blocker therapy) (11.4%), PE surgery (4.5%) or secondary prophylactic ICD implantation (1.1%), over a six-year follow-up period.

### 3.8. Risk of Bias Assessment

Regarding the RoB, the NIH quality rating was estimated as good for four studies and fair for the remaining four studies (Table 2).

Cohen’s Kappa coefficient for the agreement between the reviewers in the RoB assessment was interpreted as a substantial agreement: K = 0.80.

### 3.9. Meta-Analysis Results

**Figure 2:**
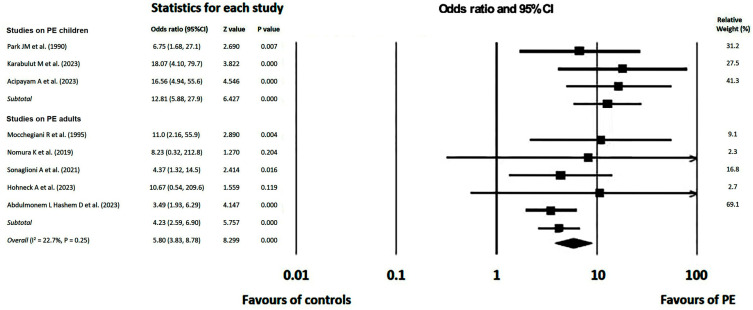
Forest plots of ORs for MVP presence in PE individuals (categorized in children and adults) vs. healthy controls across included studies [23,24,25,26,27,28,29,30].

In the pooled sample, the OR for MVP presence was significantly higher in PE individuals compared to controls (OR = 5.80, 95%CI = 3.83–8.78, Z = 8.30, *p* < 0.001). Overall, high consistency was observed in the pooled effect sizes, due to the low statistical heterogeneity among the included studies (I^2^ = 22.7%, *p* = 0.25) with regard to study design, sample size, demographics, anthropometrics, degree of anterior chest wall deformity and the specific imaging method employed for detecting MVP. Subgroup analysis revealed that MVP prevalence was approximately three-fold higher among PE children and early adolescents compared with PE adults. The forest plots of ORs for MVP presence, including 95% CIs and weighted percentages across the studies, obtained using the fixed-effects approach based on inverse-variance weighting, are illustrated in Figure 2.

**Figure 3:**
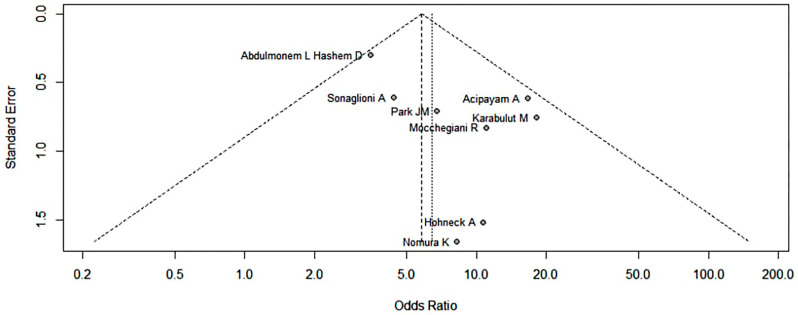
Begg’s funnel plot for publication bias detection [23,24,25,26,27,28,29,30].

Egger’s test for a regression intercept yielded a *p*-value of 0.07, indicating no publication bias and preserving the property of symmetry. In addition, Begg’s funnel plot for publication bias detection is illustrated in Figure 3.

The sensitivity analysis, performed by sequentially omitting each individual study and re-calculating the meta-analysis, supported the robustness of results. Omitting each study sequentially caused a modest variability in OR, from 5.04 (95%CI = 3.24–7.84) to 9.53 (95%CI = 5.32–17.09).

## 4. Discussion

### 4.1. Main Findings of the Present Systematic Review and Meta-Analysis

The present systematic review and meta-analysis demonstrated an increased prevalence of MVP in individuals with PE compared to healthy controls with normal chest wall conformation. The pooled prevalence of MVP among PE individuals was 40.6%; approximately one out of three PE individuals had MVP. Overall, PE individuals were nearly six times more likely to have MVP than controls. PE children and early adolescents showed a three-fold higher prevalence of MVP in comparison to PE adults. MVR due to MVP, detected in only one-fifth of PE patients, was mild or mild-to-moderate, with no evidence of hemodynamic effects. In addition, compared to controls, PE individuals were predominantly males, with (1) significantly smaller BSA; (2) RV dilatation and dysfunction; (3) SV and CO impairment, despite preserved LVEF; (4) attenuation of biventricular myocardial strain parameters, with peculiar involvement of basal segments, that are in close anatomical continuity with anterior chest wall depression; and (5) increased prevalence of isolated supraventricular and/or ventricular premature beats and NS-STT abnormalities on resting ECG.

The increased prevalence of PE among males is consistent with literature data, reporting that males are three to five times more affected than females [34]. The smaller BSA detected among PE individuals compared with controls confirms that a tall and slender build is a risk factor for PE [35].

Based on the more recent and more specific echocardiographic criteria for MVP diagnosis, which take into consideration the saddle-shaped configuration of the mitral annulus [14,36], MVP prevalence within the general population is 2–4% [31,37]. The results of the present systematic review and meta-analysis would suggest that MVP prevalence among PE individuals is approximately ten-fold higher than that observed in the general population.

MVP showed a benign course in PE individuals, as no cases of severe MVR, malignant VAs and/or SCD were reported by the included studies. These findings are consistent with literature data indicating that MVP is generally a benign condition, commonly associated with a narrow A-P thoracic diameter, isolated VPBs, mild-to-moderate MVR, a reduced burden of cardiovascular disease and a low frequency of serious complications, such as severe MMR, infective endocarditis, heart failure and atrial fibrillation [31,38,39].

### 4.2. Pathophysiological Mechanisms Underpinning the Association Between PE and MVP

The reason for the association between PE deformity and MVP is not completely understood. An embryologic explanation for this association has been proposed, since the primordia of the mitral valve undergo differentiation to their final form around the 5th to 6th week of gestation, the same period during which the vertebral column and the thoracic cage start their chondrification and ossification. A defect in growth patterns at this stage of development might affect both the mitral valve and the bony thorax [40,41]. Chest deformities are mostly a progressive process. They may be present at birth but often become more prominent during childhood, especially during periods of rapid growth such as adolescence [42,43]. The increased MVP prevalence detected by the included studies in PE children and early adolescents vs. PE adults was likely related to a greater distortion of mitral valve annulus, with consequent MVP, exerted by a deeper sternal depression during early adolescence period [27,28].

Furthermore, considering that MVP is known to be associated with a number of connective tissue disorders, such as MFS, Ehlers Danlos syndrome, periarteritis nodosa and joint laxity, and with congenital lesions such as atrial septal defects, Ebstein’s anomaly and Holt Oram syndrome, the association between PE and MVP might have a developmental or genetic basis [44]. The anatomical characteristics of the mitral valve commonly detected in PE individuals, particularly the shorter posterior leaflet and the longer anterior leaflet, that are probably present already during childhood, would confirm the possible involvement of genetic predisposition [25]. Another possible cause of MVP in PE individuals is related to the continuous mechanical stress exerted by the anterior chest wall deformity which would promote the mitral valve distortion and/or degeneration, causing the systolic billowing of one and/or both mitral leaflets above the annular plane [25]. The mechanical distortion of the mitral valve in PE patients might also be caused by the right-sided heart abnormalities commonly detected in PE individuals [45].

This mechanical hypothesis is further supported by a number of strain echocardiographic studies [46,47,48] that demonstrated a significant impairment in LV basal myocardial strain parameters in MVP individuals vs. healthy controls; the authors of these studies ascribed the deterioration of myocardial deformation indices to extrinsic compressive phenomena on cardiac chambers, rather than intrinsic myocardial dysfunction. The normal LVEF, the preserved apex-to-base gradient in LV deformation and the improvement of biventricular myocardial strain parameters after surgical correction reported in PE individuals [8] again indicated normal underlying cardiac function.

The pooled prevalence of MVP in PE individuals estimated by the two studies performed before the year 2000 [23,24] was 53%, whereas that estimated by the most recent studies [25,26,27,28,29,30] was 39%; this finding would suggest an “overdiagnosis” of MVP by the less recent studies. It is noteworthy that two studies conducted before the year 2000 assumed the planarity of mitral annulus, thus considering the superior leaflet displacement in the apical four-chamber as the diagnostic standard for MVP [49]. Conversely, the other six studies performed after the year 2000 used the new 2D echocardiographic criteria for MVP, which are more specific than the previous criteria, assuming systolic mitral annular nonplanarity [50].

### 4.3. Implications for Clinical Practice

Individuals with various degrees of PE and MVP are commonly encountered in clinical practice. The strong association between PE and MVP highlighted by the included studies would suggest that clinicians should suspect MVP in PE individuals and, vice versa, should research anterior chest wall deformity in MVP patients.

**Figure 4:**
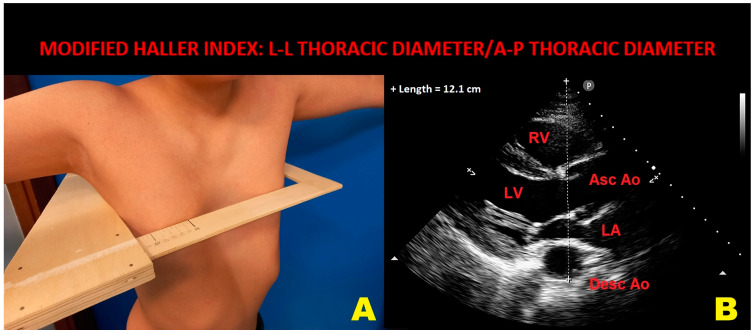
An example of MHI assessment in a PE individual. Panel (**A**): L-L thoracic diameter, measured with the individual in the standing position and with open arms by using a rigid ruler in centimeters coupled to a level (the measuring device), placed at the distal third of the sternum, at the point of maximum depression of the sternum. Panel (**B**): A-P thoracic diameter, obtained with the individual in the left-lateral decubitus position during conventional transthoracic echocardiography by placing a 2.5 mHz transducer near the sternum in the left third or fourth intercostal space, to obtain a parasternal long-axis view, and measuring the distance between the true apex of the sector and the anterior surface of the vertebral body. The vertebral body is identified by using, as a reference point, the posterior wall of the descending thoracic aorta, visualized behind the left atrium. Ao, aorta; A-P, antero-posterior; Asc, ascending; Desc, descending; LA; left atrium; L-L, latero-lateral; LV, left ventricle; MHI, modified Haller index; PE, pectus excavatum; RV, right ventricle.

Even if radiological HI is currently the standard metric for quantifying PE degree, noninvasive alternatives are needed to avoid the ionizing exposure, particularly in children with PE. In this vein, our study group developed the MHI, a nonradiological index obtained by dividing the latero-lateral (L-L) maximum external thoracic diameter (measured by a rigid ruler coupled to a level) by the A-P minor internal thoracic diameter (measured during conventional transthoracic echocardiography as the distance between the true apex of the sector and the posterior wall of the descending thoracic aorta, visualized behind the left atrium) (Figure 4).

Both thoracic diameters are measured at the end of assessment. The MHI was validated in 2018 in a comparative study of transthoracic ultrasound and chest X-Rays [51]. The validation study demonstrated narrow limits of agreement between the two measurement methods, despite a mild systematic overestimation of the new method as compared with the standard radiological HI. During the last few years, we demonstrated that PE individuals, as noninvasively defined by a MHI >2.5, not only have increased prevalence of MVP [26], but also an increased probability of being diagnosed with a false positive result on exercise stress echocardiography [52] and a significantly reduced probability of adverse cardiovascular events over a mid-term follow-up [53,54]. The basal sternal compression on cardiac chambers was considered the main causal factor for MVP genesis and for MVP-related arrhythmias and symptoms, such as atypical chest pain and palpitations. In addition, the cardiac malposition due to pectus deformity can lead to modified heart motion, cardiac rotation and tilting with “septal bounce” and dyssynchrony of myocardial wall segments, enhanced by physical exercise.

In light of these considerations, cardiologists should be aware that a number of ECG findings, such as NS-STT abnormalities or isolated extrasystoles, and/or regional wall motion abnormalities both at rest and during physical exercise and/or deterioration of myocardial deformation indices, commonly detected among MVP individuals, might be primarily related to their chest wall conformation. Accordingly, noninvasive chest shape assessment by MHI (abnormal value >2.5) or the A-P thoracic diameter (abnormal value < 13 cm) assessment where the measuring device is not available should be implemented in the clinical practice for identifying PE individuals with an increased probability of concomitant MVP and, vice versa, MVP individuals with an increased probability of concomitant pectus deformity.

Considering the low risk of adverse cardiovascular events among PE patients with MVP, further instrumental examinations such as exercise stress testing and 24 h ECG Holter monitoring may be deferred, particularly in asymptomatic individuals with mild-to-moderate MVR due to nonclassic MVP (maximal leaflet thickness <5 mm), isolated extrasystoles and the reduced burden of cardiovascular disease. Clinicians could also avoid exercise stress echocardiography and/or further unnecessary exams (such as coronary computed tomography scan and/or coronary angiography) because of the extremely low prevalence of coronary artery disease and the excellent prognosis of these individuals [53,54]. In addition, PE individuals with MVP without MR or without thickened leaflets on TTE should not undergo prophylaxis against IE [55]. Conversely, MVP patients with thickened leaflets and documented MR on TTE may benefit from antibiotics during procedures that often lead to bacteremia. Due to the increased incidence of malignant VAs and SCD observed among MVP individuals with myxomatous and bileaflet MVP and with the presence of MAD [56], PE individuals with these echocardiographic characteristics should undergo regular 24-h ECG Holter monitoring at diagnosis and during follow-up, with detailed evaluation of arrhythmias [57]. In selected cases, particularly in MVP patients with abnormal electrocardiographic or Holter findings, a multimodality imaging approach comprehensive of cardiac magnetic resonance would improve the MAD detection and better define its circumferential extent [58].

### 4.4. Limitations of the Included Studies

The main limitations of the included studies were the small sample sizes, their monocentric nature and the retrospective nature of half of them. Moreover, most studies (87.5%) did not perform a sample size calculation and used unadjusted data. Finally, data concerning medical treatment and follow-up of PE individuals were scanty. Further studies conducted on individuals with various degrees of PE and concomitant MVP are needed to determine if a narrow A-P thoracic diameter might cause a different prevalence of MVP complications in PE patients compared to individuals with normal chest shape conformation.

## 5. Conclusions

PE individuals are nearly six times more likely to have MVP than controls. MVP prevalence is three-fold higher in PE individuals during childhood and early adolescence, compared to PE adults.

Given the strong association between MVP and PE, MVP should be suspected in all individuals with anterior chest wall deformity.

The innovative MHI method should be implemented in clinical practice for identifying individuals with PE and/or MVP. This method allows a comprehensive assessment of chest wall conformation, without ionizing radiation exposure. An MHI >2.5 and an A-P thoracic diameter <13 cm are predictive of MVP presence and are generally associated with good prognosis over mid-term follow-up.

## Figures and Tables

**Table 1:** Summary and main findings of included studies. 2D, two-dimensional; BSA, body surface area; CHD, congenital heart disease; Ctrls, controls; CO, cardiac output; GCS, global circumferential strain; GLS, global longitudinal strain; GRS, global radial strain; LAD, left atrial diameter; LGE, late gadolinium enhancement; LV, left ventricular; LVEF, left ventricular ejection fraction; MAD, mitral annular disjunction; MVP, mitral valve prolapse; MVR, mitral valve regurgitation; NS-STT, nonspecific ST-segment and T-wave; PE, pectus excavatum; RV, right ventricular; RVEF, right ventricular ejection fraction; RVOT, right ventricular outflow tract; SV, stroke volume; TAPSE, tricuspid annular plane systolic escursion; TVP, tricuspid valve prolapse; VPBs, ventricular premature beats. The symbol ↔ indicates the absence of a statistically significant difference between PE individuals and controls; the symbol ↓ indicates a statistically significant reduction in the parameter in PE individuals vs. controls; the symbol ↑ indicates a statistically significant increase in the parameter in PE individuals vs. controls.

Study Name and Country	Mean Age (yrs)	Male Sex (%)	Imaging Method	MVP Rate (%)	Main Findings in PE Individuals vs. Controls
Park JM et al. (1990) [23], USA	PE = 7.6 Ctrls = 5.4	PE = 80 Ctrls = 67	M-mode echo	PE = 60 Ctrls = 18.2	↑ MVP rate in the patients older than 8 years and with severe pectus; ↑ MVR rate (20 vs. 2.6%).
Mocchegiani R et al. (1995) [24], Italy	PE = 26 Ctrls = 31	PE = 82 Ctrls = 79	2D-echo	PE = 50 Ctrls = 8.3	↓ RVOT diameter, rounded RV apex (46%), global RV dilatation (46%); ↓ RV emptying fraction; ↑ MVR rate (10.7 vs. 0%); ↑ TVP rate (11 vs. 4%).
Nomura K et al. (2019) [25], Japan	PE = 31.6 Ctrls = 58.8	PE = 56.3 Ctrls = 61.9	2D-echo	PE = 6.2 Ctrls = 0	↓ P2 scallop; ↑ A2 scallop; ↓ coaptation depths; ↑ papillary muscle tethering lengths; ↑ MVR rate (6.2% vs. 0%).
Sonaglioni A et al. (2021) [26], Italy	PE = 55.8 Ctrls = 57	PE = 60 Ctrls = 50	2D-echo	PE = 46.7 Ctrls = 16.7	↑ VPBs and NS-STT abnormalities; ↔ MVR rate (26.7 vs. 16.7%); ↓ LV and RV size, SV, CO; ↔ LVEF, E/A, E/e’, TAPSE; ↑ aortic root; ↓ LV-GLS, LV-GCS, LV-GRS, RV-GLS.
Karabulut M et al. (2023) [27], Turkey	PE = 10 Ctrls = 12	PE = 80 Ctrls = 85	2D-echo	PE = 25.6 Ctrls = 1.9	↑ MVR rate (11 vs. 3%); ↑ MVP and MVR rates with children’s age and with degree of pectus deformity.
Acipayam A et al. (2023) [28], Turkey	PE = 11.5 Ctrls = 10.9	PE = 80.6 Ctrls = 70.2	2D-echo	PE = 55.5 Ctrls = 7	↑ Cardiac malposition, MVP, MVR (25 vs. 0%), TVP (25 vs. 1.7%) and CHD rates; ↓ LAD, SV, tricuspid annulus diameter, TAPSE; ↑ ascending aorta.
Hohneck A et al. (2023) [29], Germany	PE = 29.6 Ctrls = 34.7	PE = 52 Ctrls = 52	CMR	PE = 16 Ctrls = 0	↑ A2 scallop; ↑ arrhythmias; ↑ LV volumes; ↔ LVEF, LV-GLS, LV-GCS, LV-GRS; ↑ RV volumes; ↓ RVEF, TAPSE, RV-GLS, RV-GRS; pericardial effusion (24%), LGE (12%).
Abdulmonem L Hashem D et al. (2023) [30], Canada	PE = 33.4 Ctrls = 38.1	PE = 55 Ctrls = 63	CMR	PE = 56.6 Ctrls = 27.2	↓ BSA; ↑ β-blocker therapy; ↔ LV volumes, LVEF; ↑ RV volumes; ↓ RVEF; ↑ MVP and MAD prevalence; ↔ MVR rate (24 vs. 27%).

**Table 2:** Quality assessment of included studies. Q1: Was the research question or objective in this paper clearly stated and appropriate? Q2: Was the study population clearly specified and defined? Q3: Did the authors include a sample size justification? Q4: Were controls selected or recruited from the same or similar population that gave rise to the cases (including the same timeframe)? Q5: Were the definitions, inclusion and exclusion criteria, algorithms or processes used to identify or select cases and controls valid, reliable and implemented consistently across all study participants? Q6: Were the cases clearly defined and differentiated from controls? Q7: If less than 100 percent of eligible cases and/or controls were selected for the study, were the cases and/or controls randomly selected from those eligible? Q8: Was there use of concurrent controls? Q9: Were the investigators able to confirm that the exposure/risk occurred prior to the development of the condition or event that defined a participant as a case? Q10: Were the measures of exposure/risk clearly defined, valid, reliable and implemented consistently (including the same time period) across all study participants? Q11: Were the assessors of exposure/risk blinded to the case or control status of participants? Q12: Were key potential confounding variables measured and adjusted statistically in the analyses? If matching was used, did the investigators account for matching during study analysis? Good: met 9–12 criteria; Fair: met 5–8 criteria; Poor: met 0–4 criteria. NIH, National Institutes of Health; NS, not specified.

NIH Quality Assessment Tool of Case–Control Studies Criteria Met													
Study Name	Q1	Q2	Q3	Q4	Q5	Q6	Q7	Q8	Q9	Q10	Q11	Q12	Quality
Park JM et al. [23]	Yes	Yes	No	NS	Yes	Yes	NS	Yes	Yes	NS	NS	No	6 (Fair)
Mocchegiani R et al. [24]	Yes	Yes	No	NS	Yes	Yes	NS	Yes	Yes	Yes	Yes	Yes	9 (Good)
Nomura K et al. [25]	Yes	Yes	No	Yes	Yes	Yes	NS	Yes	Yes	Yes	NS	No	8 (Fair)
Sonaglioni A et al. [26]	Yes	Yes	Yes	No	Yes	Yes	Yes	Yes	Yes	Yes	No	No	9 (Good)
Karabulut M et al. [27]	Yes	Yes	No	No	Yes	Yes	NS	Yes	Yes	Yes	NS	No	7 (Fair)
Acipayam A et al. [28]	Yes	Yes	No	Yes	Yes	Yes	NS	Yes	Yes	Yes	NS	No	8 (Fair)
Hohneck A et al. [29]	Yes	Yes	No	Yes	Yes	Yes	NS	Yes	Yes	Yes	Yes	No	9 (Good)
Abdulmonem L Hashem D et al. [30]	Yes	Yes	No	Yes	Yes	Yes	NS	Yes	Yes	Yes	Yes	No	9 (Good)

## Data Availability

Data extracted from the included studies will be publicly available on Zenodo (https://zenodo.org, accessed on 1 December 2024).

## References

[1] Janssen N., Coorens N.A., Franssen A.J.P.M., Daemen J.H.T., Michels I.L., Hulsewé K.W.E., Vissers Y.L.J., de Loos E.R. (2024). Pectus excavatum and carinatum: A narrative review of epidemiology, etiopathogenesis, clinical features, and classification. J. Thorac. Dis..

[2] Biavati M., Kozlitina J., Alder A.C., Foglia R., McColl R.W., Peshock R.M., Kelly R.E., Garcia C.K. (2020). Prevalence of pectus excavatum in an adult population-based cohort estimated from radiographic indices of chest wall shape. PLoS ONE.

[3] Jaroszewski D.E., Farina J.M., Gotway M.B., Stearns J.D., Peterson M.A., Pulivarthi V.S.K.K., Bostoros P., Abdelrazek A.S., Gotimukul A., Majdalany D.S. (2022). Cardiopulmonary Outcomes After the Nuss Procedure in Pectus Excavatum. J. Am. Heart Assoc..

[4] Fonkalsrud E.W., Dunn J.C., Atkinson J.B. (2000). Repair of pectus excavatum deformities: 30 years of experience with 375 patients. Ann. Surg..

[5] Kelly R.E., Goretsky M.J., Obermeyer R., Kuhn M.A., Redlinger R., Haney T.S., Moskowitz A., Nuss D. (2010). Twenty-one years of experience with minimally invasive repair of pectus excavatum by the Nuss procedure in 1215 patients. Ann. Surg..

[6] Haller J.A., Kramer S.S., Lietman S.A. (1987). Use of CT scans in selection of patients for pectus excavatum surgery: A preliminary report. J. Pediatr. Surg..

[7] Nuss D. (2008). Minimally invasive surgical repair of pectus excavatum. Semin. Pediatr. Surg..

[8] Chao C.J., Jaroszewski D., Gotway M., Ewais M., Wilansky S., Lester S., Unzek S., Appleton C.P., Chaliki H.P., Gaitan B.D. (2018). Effects of Pectus Excavatum Repair on Right and Left Ventricular Strain. Ann. Thorac. Surg..

[9] Raggio I.M., Martínez-Ferro M., Bellía-Munzón G., Capunay C., Munín M., Toselli L., Carrascosa P., Rodríguez-Granillo G.A. (2020). Diastolic and Systolic Cardiac Dysfunction in Pectus Excavatum: Relationship to Exercise and Malformation Severity. Radiol. Cardiothorac. Imaging.

[10] Sonaglioni A., Nicolosi G.L., Lombardo M. (2024). The relationship between mitral valve prolapse and thoracic skeletal abnormalities in clinical practice: A systematic review. J. Cardiovasc. Med..

[11] Gilbert B.W., Schatz R.A., VonRamm O.T., Behar V.S., Kisslo J.A. (1976). Mitral valve prolapse. Two-dimensional echocardiographic and angiographic correlation. Circulation.

[12] Morganroth J., Jones R.H., Chen C.C., Naito M. (1980). Two dimensional echocardiography in mitral, aortic and tricuspid valve prolapse. The clinical problem, cardiac nuclear imaging considerations and a proposed standard for diagnosis. Am. J. Cardiol..

[13] Levine R.A., Stathogiannis E., Newell J.B., Harrigan P., Weyman A.E. (1988). Reconsideration of echocardiographic standards for mitral valve prolapse: Lack of association between leaflet displacement isolated to the apical four chamber view and independent echocardiographic evidence of abnormality. J. Am. Coll. Cardiol..

[14] Freed L.A., Levy D., Levine R.A., Larson M.G., Evans J.C., Fuller D.L., Lehman B., Benjamin E.J. (1999). Prevalence and clinical outcome of mitral-valve prolapse. N. Engl. J. Med..

[15] Moher D., Liberati A., Tetzlaff J., Altman D.G., PRISMA Group (2009). Preferred reporting items for systematic reviews and meta-analyses: The PRISMA statement. PLoS Med..

[16] Ma L.L., Wang Y.Y., Yang Z.H., Huang D., Weng H., Zeng X.T. (2020). Methodological quality (risk of bias) assessment tools for primary and secondary medical studies: What are they and which is better?. Mil. Med. Res..

[17] Cochran W.G. (1950). The comparison of percentages in matched samples. Biometrika.

[18] Higgins J.P., Thompson S.G. (2002). Quantifying heterogeneity in a meta-analysis. Stat. Med..

[19] Borenstein M., Hedges L.V., Higgins J.P., Rothstein H.R. (2010). A basic introduction to fixed-effect and random-effects models for meta-analysis. Res. Synth. Methods.

[20] Lee C.H., Cook S., Lee J.S., Han B. (2016). Comparison of Two Meta-Analysis Methods: Inverse-Variance-Weighted Average and Weighted Sum of Z-Scores. Genom. Inform..

[21] Egger M., Davey Smith G., Schneider M., Minder C. (1997). Bias in meta-analysis detected by a simple, graphical test. BMJ.

[22] Thabane L., Mbuagbaw L., Zhang S., Samaan Z., Marcucci M., Ye C., Thabane M., Giangregorio L., Dennis B., Kosa D. (2013). A tutorial on sensitivity analyses in clinical trials: The what, why, when and how. BMC Med. Res. Methodol..

[23] Park J.M., Varma S.K. (1990). Pectus excavatum in children: Diagnostic significance for mitral valve prolapse. Indian J. Pediatr..

[24] Mocchegiani R., Badano L., Lestuzzi C., Nicolosi G.L., Zanuttini D. (1995). Relation of right ventricular morphology and function in pectus excavatum to the severity of the chest wall deformity. Am. J. Cardiol..

[25] Nomura K., Ajiro Y., Nakano S., Matsushima M., Yamaguchi Y., Hatakeyama N., Ohata M., Sakuma M., Nonaka T., Harii M. (2019). Characteristics of mitral valve leaflet length in patients with pectus excavatum: A single center cross-sectional study. PLoS ONE.

[26] Sonaglioni A., Nicolosi G.L., Granato A., Lombardo M., Anzà C., Ambrosio G. (2021). Reduced Myocardial Strain Parameters in Subjects with Pectus Excavatum: Impaired Myocardial Function or Methodological Limitations Due to Chest Deformity?. Semin. Thorac. Cardiovasc. Surg..

[27] Karabulut M. (2023). Increased incidence of mitral valve prolapse in children with pectus chest wall deformity. Pediatr. Int..

[28] Acipayam A., Güllü U.U., Güngör Ş. (2023). Cardiac anomalies in pediatric patients with pectus excavatum. Rev. Assoc. Med. Bras. (1992).

[29] Hohneck A., Ansari U., Natale M., Wittig K., Overhoff D., Riffel P., Boettcher M., Akin I., Duerschmied D., Papavassiliu T. (2023). Description of a new clinical syndrome: Thoracic constriction without evidence of the typical funnel-shaped depression-the “invisible” pectus excavatum. Sci. Rep..

[30] Abdulmonem L., Hashem D., Chan V.S.H., Hanneman K., Wald R.M., Thavendiranathan P., Ouzounian M., Oechslin E., Karur G.R. (2023). Association of Pectus Excavatum with Ventricular Remodelling and Mitral Valve Abnormalities in Marfan Syndrome. Can. Assoc. Radiol. J..

[31] Freed L.A., Benjamin E.J., Levy D., Larson M.G., Evans J.C., Fuller D.L., Lehman B., Levine R.A. (2002). Mitral valve prolapse in the general population: The benign nature of echocardiographic features in the Framingham Heart Study. J. Am. Coll. Cardiol..

[32] Sugimoto T., Dulgheru R., Bernard A., Ilardi F., Contu L., Addetia K., Caballero L., Akhaladze N., Athanassopoulos G.D., Barone D. (2017). Echocardiographic reference ranges for normal left ventricular 2D strain: Results from the EACVI NORRE study. Eur. Heart J. Cardiovasc. Imaging.

[33] Muraru D., Onciul S., Peluso D., Soriani N., Cucchini U., Aruta P., Romeo G., Cavalli G., Iliceto S., Badano L.P. (2016). Sex- and Method-Specific Reference Values for Right Ventricular Strain by 2-Dimensional Speckle-Tracking Echocardiography. Circ. Cardiovasc. Imaging.

[34] Fokin A.A., Steuerwald N.M., Ahrens W.A., Allen K.E. (2009). Anatomical, histologic, and genetic characteristics of congenital chest wall deformities. Semin. Thorac. Cardiovasc. Surg..

[35] Hetzer R., Siegel G., Delmo Walter E.M. (2016). Cardiomyopathy in Marfan syndrome. Eur. J. Cardiothorac. Surg..

[36] Parwani P., Avierinos J.F., Levine R.A., Delling F.N. (2017). Mitral Valve Prolapse: Multimodality Imaging and Genetic Insights. Prog. Cardiovasc. Dis..

[37] Delling F.N., Vasan R.S. (2014). Epidemiology and pathophysiology of mitral valve prolapse: New insights into disease progression, genetics, and molecular basis. Circulation.

[38] Hayek E., Gring C.N., Griffin B.P. (2005). Mitral valve prolapse. Lancet.

[39] Turker Y., Ozaydin M., Acar G., Ozgul M., Hoscan Y., Varol E., Dogan A., Erdogan D. (2009). Predictors of atrial arrhythmias in patients with mitral valve prolapse. Acta Cardiol..

[40] Bon Tempo C.P., Ronan J.A., de Leon A.C., Twigg H.L. (1975). Radiographic appearance of the thorax in systolic click-late systolic murmur syndrome. Am. J. Cardiol..

[41] Udoshi M.B., Shah A., Fisher V.J., Dolgin M. (1979). Incidence of mitral valve prolapse in subjects with thoracic skeletal abnormalities—A prospective study. Am. Heart J..

[42] Goretsky M.J., Kelly R.E., Croitoru D., Nuss D. (2004). Chest wall anomalies: Pectus excavatum and pectus carinatum. Adolesc. Med. Clin..

[43] Koumbourlis A.C. (2014). Chest wall abnormalities and their clinical significance in childhood. Paediatr. Respir. Rev..

[44] Boudoulas K.D., Pitsis A.A., Mazzaferri E.L., Gumina R.J., Triposkiadis F., Boudoulas H. (2020). Floppy mitral valve/mitral valve prolapse: A complex entity with multiple genotypes and phenotypes. Prog. Cardiovasc. Dis..

[45] Oezcan S., Attenhofer Jost C.H., Pfyffer M., Kellenberger C., Jenni R., Binggeli C., Faeh-Gunz A., Seifert B., Scharf C., Kretschmar O. (2012). Pectus excavatum: Echocardiography and cardiac MRI reveal frequent pericardial effusion and right-sided heart anomalies. Eur. Heart J. Cardiovasc. Imaging.

[46] Malev E., Zemtsovsky E., Pshepiy A., Timofeev E., Reeva S., Prokudina M. (2012). Evaluation of left ventricular systolic function in young adults with mitral valve prolapse. Exp. Clin. Cardiol..

[47] Fukuda S., Song J.K., Mahara K., Kuwaki H., Jang J.Y., Takeuchi M., Sun B.J., Kim Y.J., Miyamoto T., Oginosawa Y. (2016). Basal Left Ventricular Dilatation and Reduced Contraction in Patients with Mitral Valve Prolapse Can Be Secondary to Annular Dilatation: Preoperative and Postoperative Speckle-Tracking Echocardiographic Study on Left Ventricle and Mitral Valve Annulus Interaction. Circ. Cardiovasc. Imaging.

[48] Sonaglioni A., Nicolosi G.L., Lombardo M., Gensini G.F., Ambrosio G. (2021). Influence of chest conformation on myocardial strain parameters in healthy subjects with mitral valve prolapse. Int. J. Cardiovasc. Imaging.

[49] Morganroth J., Mardelli T.J., Naito M., Chen C.C. (1981). Apical cross-sectional echocardiography. Standard for the diagnosis of idiopathic mitral valve prolapse syndrome. Chest.

[50] Nishimura R.A., Otto C.M., Bonow R.O., Carabello B.A., Erwin J.P., Fleisher L.A., Jneid H., Mack M.J., McLeod C.J., O’Gara P.T. (2017). 2017 AHA/ACC Focused Update of the 2014 AHA/ACC Guideline for the Management of Patients with Valvular Heart Disease: A Report of the American College of Cardiology/American Heart Association Task Force on Clinical Practice Guidelines. Circulation.

[51] Sonaglioni A., Baravelli M., Vincenti A., Trevisan R., Zompatori M., Nicolosi G.L., Lombardo M., Anzà C. (2018). A New modified anthropometric haller index obtained without radiological exposure. Int. J. Cardiovasc. Imaging.

[52] Sonaglioni A., Nicolosi G.L., Rigamonti E., Lombardo M. (2022). Modified Haller index is inversely correlated with true positive exercise stress echocardiographic results. J. Cardiovasc. Med..

[53] Sonaglioni A., Rigamonti E., Nicolosi G.L., Lombardo M. (2021). Prognostic Value of Modified Haller Index in Patients with Suspected Coronary Artery Disease Referred for Exercise Stress Echocardiography. J. Cardiovasc. Echogr..

[54] Sonaglioni A., Rigamonti E., Nicolosi G.L., Lombardo M. (2021). Appropriate use criteria implementation with modified Haller index for predicting stress echocardiographic results and outcome in a population of patients with suspected coronary artery disease. Int. J. Cardiovasc. Imaging.

[55] Nishimura R.A., Carabello B.A., Faxon D.P., Freed M.D., Lytle B.W., O’Gara P.T., O’Rourke R.A., Shah P.M. (2008). ACC/AHA 2008 Guideline update on valvular heart disease: Focused update on infective endocarditis: A report of the American College of Cardiology/American Heart Association Task Force on Practice Guidelines endorsed by the Society of Cardiovascular Anesthesiologists, Society for Cardiovascular Angiography and Interventions, and Society of Thoracic Surgeons. J. Am. Coll. Cardiol..

[56] Basso C., Iliceto S., Thiene G., Perazzolo Marra M. (2019). Mitral Valve Prolapse, Ventricular Arrhythmias, and Sudden Death. Circulation.

[57] Hourdain J., Clavel M.A., Deharo J.C., Asirvatham S., Avierinos J.F., Habib G., Franceschi F., Probst V., Sadoul N., Martins R. (2018). Common Phenotype in Patients with Mitral Valve Prolapse Who Experienced Sudden Cardiac Death. Circulation.

[58] Haugaa K. (2021). Improving the imaging diagnosis of mitral annular disjunction. Heart.

